# Protein stability: a crystallographer’s perspective

**DOI:** 10.1107/S2053230X15024619

**Published:** 2016-01-26

**Authors:** Marc C. Deller, Leopold Kong, Bernhard Rupp

**Affiliations:** a Stanford ChEM-H, Macromolecular Structure Knowledge Center, Stanford University, Shriram Center, 443 Via Ortega, Room 097, MC5082, Stanford, CA 94305-4125, USA; b Laboratory of Cell and Molecular Biology, National Institute of Diabetes and Digestive and Kidney Diseases (NIDDK), National Institutes of Health (NIH), Building 8, Room 1A03, 8 Center Drive, Bethesda, MD 20814, USA; c Department of Forensic Crystallography, k.-k. Hofkristallamt, 91 Audrey Place, Vista, CA 92084, USA; d Department of Genetic Epidemiology, Medical University of Innsbruck, Schöpfstrasse 41, A-6020 Innsbruck, Austria

**Keywords:** protein stability, protein crystallization, protein disorder, crystallizability

## Abstract

Protein stability is a topic of major interest for the biotechnology, pharmaceutical and food industries, in addition to being a daily consideration for academic researchers studying proteins. An understanding of protein stability is essential for optimizing the expression, purification, formulation, storage and structural studies of proteins. In this review, discussion will focus on factors affecting protein stability, on a somewhat practical level, particularly from the view of a protein crystallographer. The differences between protein conformational stability and protein compositional stability will be discussed, along with a brief introduction to key methods useful for analyzing protein stability. Finally, tactics for addressing protein-stability issues during protein expression, purification and crystallization will be discussed.

## Abstract 2

An understanding of protein stability is essential for optimizing the expression, purification and crystallization of proteins. In this review, discussion will focus on factors affecting protein stability on a somewhat practical level, particularly from the view of a protein crystallographer.

## 1. Introduction   

The main purpose of this review is to introduce the reader to the concepts of protein stability from the viewpoint of a structural biologist, a structural biologist being defined as a scientist who determines the detailed molecular structure of a protein using methods such as crystallography, NMR spectroscopy or cryo-EM. Particular emphasis will be given to crystallographic techniques, as protein stability, or the lack thereof, represents a substantial challenge in the crystallization of many proteins. Protein stability is a wide-ranging topic including aspects of physical chemistry, thermodynamics, entropy, computational chemistry, protein folding and dynamics. For the purposes of this review, many of the computational and theoretical aspects are skipped over and the reader is referred to other excellent reviews on this topic (Compiani & Capriotti, 2013 ▸; Lazaridis & Karplus, 2002 ▸).

Stability is the potential of a pattern to survive over time, and therefore is integral to our understanding of biological systems and their evolution (Schrödinger, 1945 ▸). Clearly, the exact meaning of a ‘pattern’ for a protein molecule is somewhat vague, but we know that processes such as protein unfolding, denaturation, degradation, conformational change, enzymatic modification and proteolytic cleavage may transform this ‘pattern’. These transformations are generally considered, or analyzed, with respect to the integrity of the primary and conformational structure of the fully folded protein. Additionally, protein stability means different things to different people. For example, a pharmacologist, biotechnologist or food scientist may primarily consider the half-life of a protein’s activity as a measure of its stability. However, a protein chemist or a structural biologist may concern themselves with changes in the primary, secondary, tertiary or quaternary structure of a protein as a measure of its stability. Again, for the purposes of this review, we will focus on the structural aspects of protein stability and will refer the reader to other excellent reviews on protein stability from a pharmacological and biotechnological perspective (Hall, 2014 ▸).

We will first discuss protein stability as a fundamental prerequisite for crystallization (§[Sec sec2]2) and then some important aspects of stability on a higher, structural level (see §[Sec sec3]3). At this stage it is important to discuss the differences between thermo­dynamic protein stability and conformational protein disorder, especially given some of the unique parameters that structural biologists use to describe and analyze their structures. For example, NMR spectroscopists often report root-mean-squared deviation (r.m.s.d.) values between their ensemble structures, whereas crystallographers report *B* factors as a measure of the positional uncertainty in a given protein crystal structure model. Both of these parameters represent displacements and disorder within a structure and can be reflective of the level of conformational stability. We will then discuss some important factors to consider when expressing and purifying proteins for structural studies. Structural genomics efforts have alleviated many of the bottlenecks of a traditional structure-determination pipeline, but researchers are still all too aware of the difficulties of expressing and purifying challenging protein targets. Careful consideration of the primary structure, construct design, expression conditions and hosts cells can all be used to mitigate many of the protein-stability issues observed during expression and purification (see §[Sec sec4]4). We will then discuss some common methods used to analyze protein stability, with a focus on methods routinely used to asses protein stability, including protein melting temperature analysis (*T*_m_), NMR and cryo-EM (see §[Sec sec5]5).

## 2. Stability is a fundamental prerequisite for crystallization   

**Figure 1:**
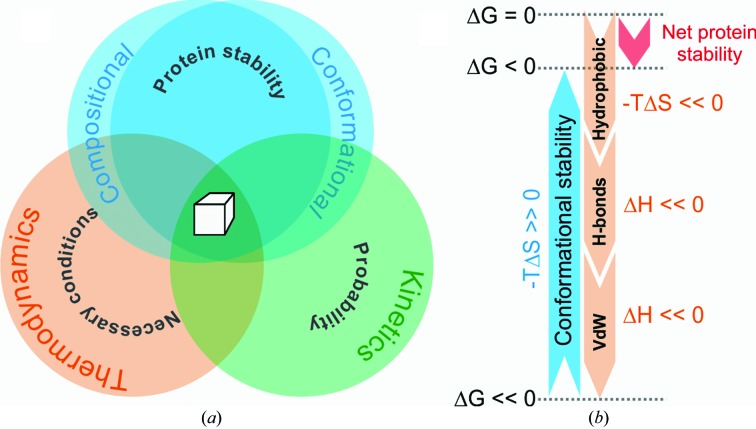
Factors influencing protein stability. (*a*) Protein *compositional* stability and *conformational* stability as key determining factors for successful crystallization. The stability properties of the protein determine whether the process of crystal formation is possible. Thermodynamics establish the necessary conditions for crystallization, and the kinetics and dynamics of the processes determine whether a possible scenario actually becomes reality. Only if all of the parameters are satisfied will crystal formation proceed. Figure adapted from Rupp (2015 ▸). (*b*) The marginal net stability of a folded protein is highlighted with respect to the contributing factors; the overwhelming lack of *conformational* stability is only marginally balanced by the contribution of van der Waals (VdW), hydrogen-bonding (H-bonds) and hydrophobic forces. Figure adapted from http://bit.ly/1L921Oi.

Biomolecular crystallization can be described as the self-organization of macromolecules into a translationally periodic arrangement with long-range order. In order to achieve this goal optimally, the moieties within each asymmetric unit of a crystal should be of the same kind and of the same shape. If a protein cannot form such stable entities *per se*, a fundamental and primary requirement for crystallization is not met, and no effort to find suitable thermodynamic and kinetic conditions will lead to crystals of such a protein construct (Fig. 1 ▸ *a*).

It is important to note that from a crystallization perspective, there are at least two major flavors of protein stability: *compositional* stability and *conformational* stability (Table 1 ▸). The crystallographer must carefully assess both types of stability in order to enable crystallization of the target protein.

### 2.1. Compositional stability   

During the processes of crystallization it is essential to maintain the same species within the crystallization experiment; there needs to be some form of *compositional* stability. On a simple level this means that the protein molecules must have the same chemical makeup. The chemical homogeneity of a sample can often be determined using mass spectrometry or an SDS–PAGE gel. Compositional homogeneity is typically compromised by post-translational modifications, such as glycosylation and proteolysis, which can affect the primary structure of the protein molecules and generate compositional variability (see §3.1). Because protein crystallization takes time, the primary requirement for compositional stability must be maintained over a period of time, and preferably within a reasonable range of environmental conditions. It is important to note that there is no such thing as absolute stability. For example, a protein that is compositionally stable enough to produce a single band on an SDS–PAGE gel may still not be stable enough over the timeframe of a crystallization experiment.

### 2.2. Conformational stability   

Assuming that the protein sample has a degree of *compositional* homogeneity, it will still likely not crystallize unless it possesses *conformational* stability. A large number of proteins fall into the category of conformationally disordered proteins displaying little or no conformational order (Longhi *et al.*, 2010 ▸). A protein with substantial disordered regions, or separate domains exhibiting dynamic variability, will be less likely to self-organize into a crystal. This can be the case even if the sample has perfect *compositional* stability. The strict requirement for limited *conformational* variability is a unique problem that a crystallographer faces when trying to crystallize a protein sample. The problem is confounded by the fact that the conformation of flexible regions of a protein is a context-driven property. For example, conformations may be quite different in the cellular context, in an NMR tube or in a macromolecular crystal. While structural methods can be used to probe conformational homogeneity, it is important to realise that the results are only meaningful within the context and conditions of that particular method (see §5). For example, analysis of conformational stability and dynamics is often limited using crystallographic methods as the crystal packing can hinder such movements. In these cases NMR solution methods can provide complementary information.

Structures determined using X-ray crystallography provide limited information regarding the dynamics of the protein structure. Nonetheless, some dynamics information is included in the atomic model in the form of the atomic displacement parameter (ADP) or *B* factor. The *B* factor is expressed in units of Å^2^ and is essentially a statistical measure describing the probability of finding an atom at that particular mean position in the structure (Willis & Pryor, 1975 ▸). If the *B* factor of a particular atom is high then it suggests that the certainty of finding the atom at that position in the structure is low. Atoms in regions of high *B* factor can be displaced as a result of dynamic disorder of the polypeptide chain or as a result of short-range or long-range disorder within the crystal. Such flexible or dynamic regions can often be identified in a crystal structure and engineered out at the cloning stage to produce protein samples with better conformational stability (see §§[Sec sec4.1]4.1 and [Sec sec6.1]6.1). Not only do such modifications result in better protein stability during expression and purification, but they also increase the probability that the molecules will pack within the crystal lattice in a more orderly fashion. As a consequence, such efforts often result in better diffracting crystals and higher-resolution X-ray data.

Comparison of ensembles of structures, as typically generated by NMR spectroscopy, can be used to provide a measure analogous to the crystallographic *B* factor in the form of a root-mean-squared deviation (r.m.s.d.) between corresponding atoms of the ensemble members. This measure can be used to assess the flexibility, dynamics, disorder or stereochemical variability across a set of structural models. The r.m.s.d. value is complementary to the crystallographic *B* factor and because the structure is in solution it is not perturbed or influenced by crystal packing (Sikic & Carugo, 2009 ▸).

A large class of proteins, referred to as intrinsically dis­ordered proteins (IDPs), contain significant levels of conformational disorder and, in some cases, have no discernable three-dimensional structure at all. It is estimated that ∼40% of all human proteins contain at least one disordered segment and ∼25% are completely disordered (Uversky & Dunker, 2010 ▸). These proteins have largely been avoided by the crystallographic community owing to the expected difficulties in crystallizing them. However, NMR techniques have been central to unraveling how these unstructured proteins function. Such studies have led to a paradigm shift in our understanding of protein structure and function (Wright & Dyson, 1999 ▸). Traditional theory dictates that proteins function by adopting a rigid, preformed structure that binds to a target ligand or protein in a fashion analogous to a lock and key. However, NMR studies on IDP proteins such as CREB, p53 and 14-3-3 have revealed that these disordered regions allow plasticity and flexibility and often only form structure upon binding of the partner protein (Oldfield *et al.*, 2008 ▸; Sugase *et al.*, 2007 ▸; Mujtaba *et al.*, 2004 ▸). These so-called ‘hub proteins’ are capable of interacting with many different protein partners in a context-sensitive manner, and this is only possible as a result of the plasticity and initial lack of conformational stability. High-resolution crystal structures of complexes of these vital ‘hub proteins’ will be essential for understanding their role in human diseases such as Parkinson’s disease and Alzheimer’s disease (Wang *et al.*, 2011 ▸). Although the poor conformational stability of these proteins poses challenges for the protein crystallographer, in a cellular context IDPs offer many advantages over more traditional single-function folded proteins, including the ability to bind to many different protein partners (Liu & Huang, 2014 ▸).

In addition to IDPs, many proteins contain aggregation-prone regions (APRs) that typically contain a run of 5–15 amino acids with a propensity for forming extended β-sheet structures. For example, APR segments are observed in β_2_-microglobulin and are responsible for aggregation into amyloid fibers in diseases such as amyloidosis (De Baets *et al.*, 2014 ▸). Another group of proteins referred to as intrinsically insoluble proteins (IIPs) are completely insoluble and cannot be refolded in traditional buffer solutions (Goyal *et al.*, 2015 ▸; Liu & Song, 2009 ▸). For example, naturally occurring mutants of SH3, such as V22-SH3, are insoluble in the presence of ions, but they can be resurrected and solubilized in pure water, allowing further study of the unstructured proteins in solution using NMR spectroscopy (Liu & Song, 2009 ▸).

## 3. Stability of the protein on a structural level   

One simple way of conceptualizing protein stability from a structural perspective is to consider stability at each level of protein structure: primary structure, secondary structure, tertiary structure and quaternary structure (Table 2 ▸). Protein stability with respect to each of the structural levels will now be discussed in turn. Wherever possible, we will emphasize aspects of particular importance to the structural biologist, with a particular focus on protein crystallization.

### 3.1. Primary structure   

The primary structure of the protein, or the sequence of the amino acids in the polypeptide chain, can be modified in several ways by post-translational modifications (PTMs). PTMs result in alteration of the structure and function of a protein and for this reason are central to any discussion of protein stability. As discussed above (see §[Sec sec2]2), PTMs can affect both *compositional* stability, as the modifications may be non-uniform or incomplete, and also *conformational* stability, as the modifications may affect protein disorder and dynamics. This is illustrated by glycoproteins, which are often not uniformly glycosylated at all possible glycosylation sites, therefore leading to compositional heterogeneity. Furthermore, complex hydrocarbon chains tend to have a greater degree of conformational freedom. This conformational freedom results in an increase in disorder on the protein surface, while at the same time shielding polar or charged residues on the protein surface required for intermolecular crystal contact formation (see §[Sec sec6.6]6.6). Although the heterogeneity of glycosylation tends to impair crystallization, its variability can have important functional implications for a protein.

PTMs are the result of many different changes to the primary structure of a protein, including proteolytic processing, protein splicing and the addition of other functional groups to the amino acids. PTMs are often used for targeting of the protein to a specific region of the cell or modification of the activity or specificity of an enzyme. For example, functional groups such as myristate, palmitate, isoprenoid and glycosylphosphatidylinositol (GPI) are often attached to the protein and used for targeting of the protein to the membrane (Chatterjee & Mayor, 2001 ▸). Other functional groups such as carboxylate (Walker *et al.*, 2001 ▸), ethanolamine phospho­glycerol (Whiteheart *et al.*, 1989 ▸) and hypusine (Park *et al.*, 2010 ▸) can be added to proteins to regulate their activity. Additionally, larger peptides and proteins can also be covalently added to proteins, including ubiquitin (Komander & Rape, 2012 ▸), SUMO (Hay, 2005 ▸), ISG15 (Malakhova *et al.*, 2003 ▸), PUP (Striebel *et al.*, 2014 ▸) and NEDD (Rabut & Peter, 2008 ▸). Of the 821 182 proteins that were experimentally analyzed by Khoury and coworkers, the top ten observed PTMs are phosphorylation (58383), acetylation (6751), N-linked glycosylation (5526), amidation (2844), hydroxylation (1619), methylation (1523), O-linked glycosylation (1133), ubiquitylation (878), pyrrolidone carboxylic acid (826) and sulfation (504) (http://bit.ly/1jdfXR8; Khoury *et al.*, 2011 ▸). Key methods used to analyze and identify changes at the primary-structure level include mass spectrometry and Eastern and Western blots (Liu *et al.*, 2014 ▸; Towbin *et al.*, 1979 ▸; Table 2 ▸).

It is important to note that many PTMs play a role in stabilizing proteins, particular with respect to the half-life and turnover of the protein within the cell. For example, PTMs such as ubiquitination target proteins to the proteasome for degradation and recycling, therefore directly affect the half-life of the protein and its stability within the cell (Komander & Rape, 2012 ▸). A myriad of other PTMs exist, including acyl­ation, alkylation, arginylation, butyrylation, malonylation, ADP-ribosylation, iodination, oxidation, succinylation, *S*-nitrosylation, *S*-glutathionylation and glycosylation. Currently, just under 500 PTMs have been identified in the SWISS-PROT and TrEMBL databases (for a full list, see http://bit.ly/1P6Rbj3). All of these modifications play a role in the structure and the function of the target protein. However, some PTMs, such as proteolytic cleavage and protein splicing, significantly influence protein structure at the primary level and can lead to drastic changes in *compositional* stability.

Protein splicing occurs in proteins called inteins (or protein introns), which are a large class of self-cleaving proteins found in all domains of life (Paulus, 2000 ▸; Novikova *et al.*, 2014 ▸). One of the first examples identified was the VMA1 protein, a yeast vacuolar membrane H^+^-ATPase, which was shown to undergo protein splicing (Hirata *et al.*, 1990 ▸). Protein splicing is a naturally occurring process analogous to the splicing of introns from RNA. A precursor polypeptide is processed into a mature and functional protein. The intein is autocatalytically excised from the precursor protein and the flanking exteins are ligated together, producing two new polypetides (Mills *et al.*, 2014 ▸). Inteins are of great importance for the stability of proteins, but they are also of interest from a protein-engineering perspective (Aranko *et al.*, 2014 ▸). For example, inteins can be used for the preparation of isotope-labeled proteins for NMR spectroscopy, for site-specific fluorescent labeling and as self-cleaving affinity purification tags such as cSAT and intein-CDB (commercially available as the IMPACT system from NEB; Chong *et al.*, 1997 ▸; Volkmann & Iwaï, 2010 ▸; Lin *et al.*, 2015 ▸; see §[Sec sec4.1]4.1).

### 3.2. Secondary structure   

Protein secondary structure is the localized three-dimensional structure of the polypeptide chain. Secondary structure can be described in terms of the pattern of hydrogen bonding between amide H atoms and carbonyl O atoms of the backbone (Pauling *et al.*, 1951 ▸) or by the stereochemistry adopted by the polypeptide backbone (Ramachandran *et al.*, 1963 ▸). On a somewhat simplified level, the primary driving forces behind the formation of secondary structure, and in turn tertiary structure, are hydrogen bonding and hydrophobic interaction (Pace, Scholtz *et al.*, 2014 ▸; see §[Sec sec3.3]3.3).

The α-helix is the predominant type of secondary structure, accounting for approximately one-third of all secondary-structure elements (Stickle *et al.*, 1992 ▸). Analysis of the first crystal structures suggested that certain residues including alanine, leucine and glutamate are found frequently in α-helices. In contrast, other residues such as proline, glycine and aspartic acid are found less frequently (Davies, 1964 ▸; Prothero, 1966 ▸; Guzzo, 1965 ▸). This information has been used to develop many algorithms for the prediction of protein secondary structure, including the popular Chou and Fasman method (Chou & Fasman, 1974 ▸). Secondary-structure propensity data have been expanded using mutagenesis data, and tables of α-helical (Pace & Scholtz, 1998 ▸) and β-sheet (Smith *et al.*, 1994 ▸) propensity have been compiled.

One overwhelming consensus of the amino-acid propensity rules is the destabilizing effect that proline has on the α-helix (ΔΔ*G* of 3.16 kcal mol^−1^ *cf.* alanine at 0 kcal mol^−1^; Pace & Scholtz, 1998 ▸; see §[Sec sec3.3]3.3). This destabilization is a result of the missing backbone amide H atom, which prevents proline from participating in stabilizing hydrogen bonding. Additionally, the bulky cyclic side chain of proline results in a ∼30% kink in the α-helix backbone as a result of steric hindrance (Richardson, 1981 ▸; Yun *et al.*, 1991 ▸). Glycine has the next lowest propensity for forming α-helices as a result of enhanced conformational flexibility upon folding to form an α-helix (Hermans *et al.*, 1992 ▸). It is important to note that many of these secondary-structure propensities are highly context-dependent. For example, proline occurs widely in the transmembrane helices of integral membrane proteins and has been shown to have a stabilizing effect on α-helices in such environments (Li *et al.*, 1996 ▸).

Clearly, such findings are in support of the hypothesis that the stability of the folded protein is largely dictated by the amino-acid composition and, as such, the primary structure results in a unique, kinetic minimum of free energy as first suggested by Anfinsen (1973 ▸). These simple principles have been expanded into complex algorithms that can be used to design both stable α-helices and β-sheets (Jiménez, 2014 ▸; Yakimov *et al.*, 2014 ▸). Furthermore, comparative modeling can be used to design proteins with a greater degree of thermal stability, and similar models can be used to predict the crystallizability of a protein (Olson *et al.*, 2015 ▸; Smialowski & Frishman, 2010 ▸; see §[Sec sec6]6).

### 3.3. Tertiary structure   

The tertiary structure of a protein is the overall shape, or fold, adopted by the polypeptide chain. Many factors affect the process of protein folding, including *conformational* and *compositional* stability, cellular environment including temperature and pH, primary and secondary structure, solvation, hydrogen bonding, salt bridges, hydrophobic effects, van der Waals (vdW) forces, ligand binding, cofactor binding, ion binding, chaperones and PTMs, to name just a few.

The *conformational* stability of the polypeptide chain results in a significant entropic penalty (−*T*Δ*S* >> 0), and under normal cellular conditions a folded protein is only marginally stable (∼10 kcal mol^−1^ for a 10 kDa protein; Fig. 1 ▸ *b*). In order to overcome this entropic penalty, all of the other factors influencing protein folding must outweigh the loss of conformational entropy (Dill, 1990 ▸). A series of studies by Pace and coworkers have recently quantified some of these influences (Pace *et al.*, 2011 ▸; Pace, Fu *et al.*, 2014 ▸; Pace, Scholtz *et al.*, 2014 ▸). These studies suggest that the hydrophobic effect contributes ∼60% to the stability of the protein and hydrogen bonding contributes ∼40% (Pace *et al.*, 2011 ▸). Specifically, the burial of a single methyl group contributes ∼1.1 kcal mol^−1^ to net protein *stability* and loss of its conformational entropy contributes ∼2.4 kcal mol^−1^ to net protein *instability* (Pace *et al.*, 2011 ▸). The net contribution of hydrogen bonding to overall protein stability is also ∼1.1 kcal mol^−1^ and is largely independent of the size of the protein (Stickle *et al.*, 1992 ▸; Pace, Scholtz *et al.*, 2014 ▸). However, in contrast, hydrophobic interactions typically contribute less to the stability of small proteins (Pace *et al.*, 2011 ▸; Pace, Fu *et al.*, 2014 ▸).

The stability of the protein fold is of particular interest for the design of thermally stable proteins for industrial uses such as biofuel production and as proteases for laundry detergents. Thermophilic organisms such as *Thermotoga maritima*, which thrives in hot deep-sea vents in the Sargasso Sea, require proteins that maintain fold and structure under such extreme conditions. The study of these thermophilic proteins suggests that the protein structures are similar to their mesophilic counterparts and thermal stability is inferred by subtle changes in the amino-acid composition. On comparing thermo­philic proteins with their mesophilic counterparts, certain patterns are observed including an increase in the number of salt bridges, an increase in hydrophobicity and an increase in the number of aromatic residues (Dekker *et al.*, 1991 ▸; Tanner *et al.*, 1996 ▸; Zhou *et al.*, 2008 ▸; Fields *et al.*, 2015 ▸; Somero, 2004 ▸).

**Figure 2:**
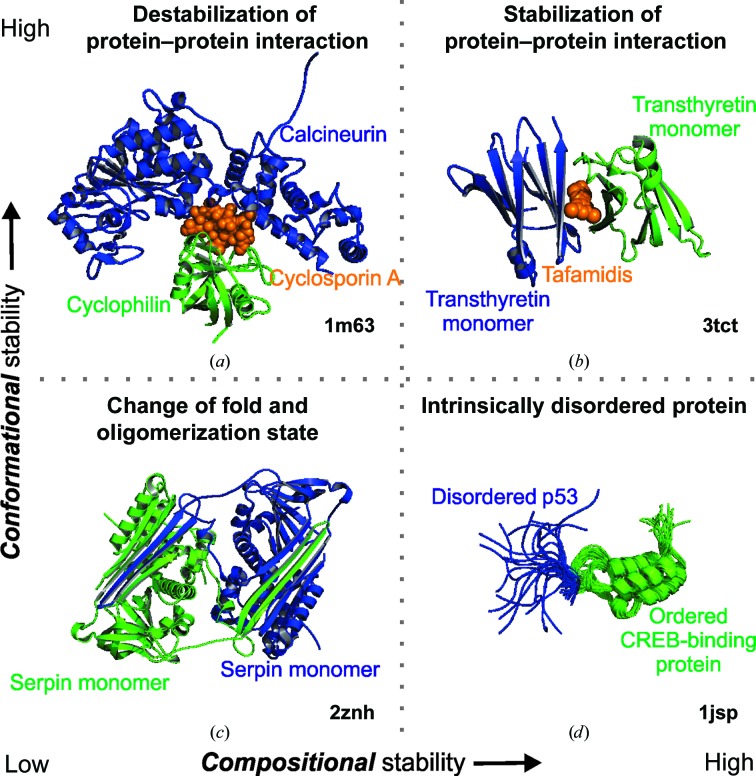
Matrix of examples of protein stability and disorder. (*a*) Examples of proteins with high *conformational* stability include the protein–protein destabilizing compound cyclosporin in complex with calcineurin and cyclophilin (Huai *et al.*, 2002 ▸) and (*b*) the protein–protein stabilizing drug Tafamidis in combination with transthyretin (Bulawa *et al.*, 2012 ▸). (*c*) Examples of proteins with low *conformational* stability include the serpins, which undergo large changes in fold and oligomerization state (Yamasaki *et al.*, 2008 ▸), and (*d*) intrinsically disordered proteins (IDPs) such as the tumor suppressor protein p53 (Mujtaba *et al.*, 2004 ▸).

In contrast to the IDPs discussed above (see §[Sec sec2.2]2.2), the stability of the protein tertiary structure is often considered to be essential for the maintenance of protein function. However, many proteins undergo an overall change of protein fold as part of their mechanism of action. For example, serine protease inhibitors (serpins) undergo a transformation from a long-term stable native form (S, stressed) into a more stable folded form (R, relaxed) upon interaction with the proteinase (Whisstock & Bottomley, 2006 ▸; Whisstock *et al.*, 2000 ▸). These structural rearrangements include the insertion of a loop into the center of a core β-sheet or the insertion of a β-strand to form a domain-swapped dimer that can initiate polymerization (Mottonen *et al.*, 1992 ▸; Yamasaki *et al.*, 2008 ▸; see Fig. 2 ▸ *c*). Large conformational changes such as this are commonplace and are observed in many proteins including influenza virus hemagglutinin, lymphotactin, Mad2 spindle checkpoint protein and chloride intracellular channel 1 (CLIC1; Bryan & Orban, 2010 ▸). Therefore, it is important that any discussion on stability carefully considers the mechanism of the protein under study, as some protein folds are designed to be inherently unstable.

### 3.4. Quaternary structure   

Quaternary structure is the arrangement of the folded protein subunits into a multi-subunit complex. The stability of such complexes is of importance for the regulation of allostery and cooperativity, which often results from the conformational changes within individual polypeptide chains. One of the classic models used to describe allosteric transitions in proteins is the Monod–Wyman–Changeux (MWC) model (Monod *et al.*, 1965 ▸). In this model proteins may exist in one of two states: tense (T) and relaxed (R). One of the key features of this model is that ligands may bind to either the T or R state with equal affinity, but if the R state is preferred then the affinity will be increased. However, if binding to the T state is favored then the affinity is decreased and the substance is described as an allosteric modulator. One of the best-studied examples is hemoglobin, with the R state representing deoxyhemoglobin and the T state representing oxyhemo­globin (Brunori, 2014 ▸; Ronda *et al.*, 2013 ▸). As discussed above for IDPs and metastable proteins (see §§[Sec sec2.2]2.2 and [Sec sec3.3]3.3), it is not sufficient to consider protein stability in isolation from function. Many proteins undergo large conformational changes involving both secondary and tertiary structure, and each state may have a different *conformational* or *compositional* stability (Fig. 2 ▸).

Modulation of the quaternary structure, and more specifically the protein–protein interactions responsible for quaternary structure, has long been a goal for the pharmaceutical industry. Such efforts present considerable challenges as a result of the large surface areas involved. Small molecules that *destabilize* protein–protein interactions have been demonstrated, but *stabilizing* examples are somewhat scarce (Giordanetto *et al.*, 2014 ▸; Wells & McClendon, 2007 ▸). Destabilizing examples include the Abbott drug Navitoclax, which destabilizes the interaction between the anti-apoptotic protein Bcl-2 and Bad/Bid/Bak (Oltersdorf *et al.*, 2005 ▸), and the Roche drug Nutlin-3, which inhibits the interaction between the tumor suppressor p53 and MDM2 (Secchiero *et al.*, 2011 ▸). Examples of compounds that stabilize protein–protein interactions include natural products such as cyclosporin A, which stabilizes the interaction between calcineurin and cyclophin (Huai *et al.*, 2002 ▸), and the drug Tafamidis, which binds to a pocket at the interface of the transthyretin dimer (Bulawa *et al.*, 2012 ▸) (see Figs. 2 ▸ *a* and 2 ▸ *b*, respectively). In the case of Tafamidis, stabilization of the dimerized form of transthyretin prevents the aggregation and misfolding which has been shown to be the mechanism leading to transthyretin amyloid­osis diseases such as peripheral neuropathy.

## 4. Protein stability during protein expression and purification   

The stability of the protein during the course of expression and purification is often an issue. In order to obtain sufficient quantities of protein for crystallization screening, formulation, vaccine development or therapeutic use, it is essential that intact, stable and folded protein is produced. Many proteins are unstable, unfolded or proteolytically cleaved under the conditions used for protein expression; again it is important to emphasize that these factors can lead to poor stability on both the *conformational* and *compositional* levels (see §[Sec sec2]2 and Table 1 ▸). Factors giving rise to poor protein stability during expression and purification may include the primary structure of the protein, the construction of the recombinant expression plasmid, the temperature and the expression medium used and the toxicity of the protein to the host organism (Table 3 ▸). Therefore, there are many factors to test during expression and purification, and combinatorial design approaches are often used, in combination with high throughput methods to find the appropriate combination of conditions (Papaneophytou & Kontopidis, 2014 ▸). The use of advanced genetic engineering methods to modify both cells and expression plasmids is covered in more detail in reviews such as Sørensen & Mortensen (2005 ▸).

### 4.1. Construct design, sequence, expression tags and protein stability   

The design of the expression construct is one of the primary decisions that a structural biologist must make to ensure the efficient expression and purification of the target protein. For example, the *compositional* stability of a protein can be severely affected by the presence of protease cleavage sites within the target protein. These sites can result in cleavage of the target protein by endogenous proteases produced by the expression host. Proteolysis can be mitigated by the removal of cleavage sites from the expression construct during recombinant assembly. For example, such an approach was used to remove two protease sites from a malarial vaccine candidate that was proteolytically degraded by endogenous KEX2 protease during expression. Removal of these sites enabled the production of full-length protein on a large scale (Spiegel *et al.*, 2015 ▸). Additionally, the primary structures of some proteins are inherently unstable, with unusually short half-lives. For example, proteins containing sequences rich in proline, glutamate, serine and threonine (PESTs) often have half-lives of less than 2 h (Rogers *et al.*, 1986 ▸). It is suggested that PEST sequences target the protein for intracellular degradation *via* the proteasome machinery (Spencer *et al.*, 2004 ▸) or *via* more traditional proteolysis pathways utilizing calpain (Shumway *et al.*, 1999 ▸). Furthermore, the N-end rule is a strong predictor of protein half-life *in vivo*. For example, if the N-terminal residue of a protein is methionine, serine, alanine, glycine, threonine, valine or proline the half-life is stabilized (>20 h). In contrast, if the N-terminal residue is phenylalanine, aspartate, lysine or arginine the half-life of the protein is destabilized (<3 min; Bachmair *et al.*, 1986 ▸). Proteins destabilized in this way are targeted for degradation *via* the ubiquitination pathway; therefore, the N-end rule is of no concern when using bacterial expression systems. It is important to note that the N-terminal residue is ‘masked’ by the inclusion of an N-terminal affinity purification tag, as is typically used in most laboratories today.

In addition to the half-life stabilizing effects of affinity purification tags, they are also of considerable interest for improving the solubility of a target protein (Amarasinghe & Jin, 2015 ▸; Wood, 2014 ▸). This is particularly true for maltose-binding protein (MBP), which has a strong effect in solubilizing the protein to which it is attached. MBP has also been shown to promote the correct folding of the protein target to which it is attached, suggesting that it can act as a form of molecular chaperone (Kapust & Waugh, 1999 ▸). Other chaperones that can be used to assist protein stability and folding during expression include DnaK and GroEL (Kyratsous & Panagiotidis, 2012 ▸). Some proteins are simply not stable in the cytoplasm and they can be redirected to other compartments of the host cell using an affinity tag. For example, the pMal vector system (NEB) incorporates the malE signal sequence and can be used to direct the protein of interest across the plasma membrane and into the periplasm. This has the added advantage of keeping the target protein away from cytoplasmic proteases during subsequent purification steps, thus further enhancing the *compositional* stability.

Self-cleaving affinity purification tags can be applied to carefully control the *compositional* stability. Traditional affinity-tag removal procedures often use proteases, such as thrombin or factor Xa, which can result in nonspecific degradation of the target protein. However, the use of highly specific self-cleaving tags prevents this issue as exogenous protease addition is not required. In addition to the self-cleaving intein-based tags discussed above (see §[Sec sec3.1]3.1), it has also been reported that nickel ions can be used to cleave an affinity tag. In the example of the GmSPI-2 inhibitor structure the peptide bond preceding the serine or threonine residue in the (S/T)*X*HZ motif was cleaved by nickel ions (Kopera *et al.*, 2014 ▸; Krężel *et al.*, 2010 ▸).

### 4.2. Expression conditions and protein stability   

In order to ensure proper protein folding and stability, it is essential that the expression host is provided with the necessary prosthetic groups, cofactors and ligands as required by the target protein. Many of these are provided by the expression medium and are scavenged by the host cells during the course of expression. However, some cofactors and prosthetic groups cannot be synthesized by the host cell and others are not available in sufficient quantities. For example, heme incorporation is often low when heme-containing proteins are expressed in *Escherichia coli*. In these cases the expression medium must be supplemented with δ-aminolevulinate in order to achieve satisfactory levels of heme incorporation (Kery *et al.*, 1995 ▸). Additionally, the solubility of the protein can also be significantly improved during expression by the addition of additives such as trehalose, glycine betaine, mannitol, l-arginine, potassium citrate, CuCl_2_, proline, xylitol, NDSB 201, CTAB and K_2_PO_4_ (Leibly *et al.*, 2012 ▸; see §[Sec sec6.2]6.2 for more on buffer screening).

Varying the temperature at which the expression is carried out can also be used to control protein stability and solubility. For example, cold-shock induction systems such as pCold (Takara/Clontech) can be used to improve the overall stability of the target protein (Qing *et al.*, 2004 ▸). As an added benefit, at lower temperatures, cell proliferation is halted and the expression of endogenous proteins such as proteases is reduced. Therefore, the target protein is further protected from degradation and purity is improved. To assist in low-temperature expression, cold-adapted *E. coli* cells, for example ArcticExpress (Agilent Technologies), have been developed. These cells co-express the cold-adapted chaperonins Cpn10 and Cpn60 from the psychrophilic bacterium *Oleispira antartica* (see §[Sec sec4.3]4.3 for more on host-cell selection).

### 4.3. Host cells and protein stability   

The choice of the host cells that are used for the expression of recombinant proteins has an important influence on protein stability. For example, the protein may be toxic to the host cell or the protein may be cleaved by endogenous proteases made by the cell. Clearly, such issues can be a primary source of *compositional* instability in proteins. Toxicity can be controlled by tight regulation of the expression level using promoters that respond in a concentration-dependent manner to the inducer. Examples of tightly controlled expression vectors include pBAD, which responds to the inducer l-arabinose (Guzman *et al.*, 1995 ▸). The background expression of proteases (and proteins in general) can also be controlled using plasmids that express T7 lysozyme, such as pLysS. T7 lysozyme is a natural inhibitor of T7 RNA polymerase, the promoter utilized in the pET vector system, and can be used to reduce background levels of protein expression (Studier, 1991 ▸). Background levels of protease activity can also be reduced using OmpT^−^ bacterial strains, which do not express the outer membrane aspartyl protease. Such systems are commercially available as BL21 Star strains of *E. coli* (Invitrogen/ThermoFisher Scientific).

Host-cell selection is particularly important when expressing mammalian proteins in bacterial cells, as the codon usage between the organisms is different. For example, the AGA codon for arginine is particularly rare in *E. coli* and can result in premature chain termination, frame-shifting and incorrect amino-acid insertion (Calderone *et al.*, 1996 ▸, Kane, 1995 ▸). This issue can be addressed in a number of ways, including the generation of a synthetic gene reflecting the codon usage of the host organism or by co-transformation of the host with a plasmid that can provide the tRNA of the missing codons (*e.g.* CodonPlus, Stratagene and pRARE; EMD Millipore/Novagen; Dieci *et al.*, 2000 ▸; Fu *et al.*, 2007 ▸). Competent *E. coli* BL21 cells containing the pRARE plasmid are commercially available under the trade name Rosetta. These cells have been used to optimize the expression of many human proteins in *E. coli*. For example, the Swedish Human Protein Atlas project has been successful in improving both the level of expression and the purity of proteins using the Rosetta *E. coli* strain (Tegel *et al.*, 2010 ▸).

Finally, mammalian expression systems such as Chinese hamster ovary (CHO) cells (Fischer *et al.*, 2015 ▸) and human embryonic kidney cells (*e.g.* HEK 293T and 293F; Nettleship *et al.*, 2015 ▸) are often essential for the expression of mammalian or human proteins (for a discussion of the merits of the various expression systems, see Brondyk, 2009 ▸). In addition to addressing the codon-usage issue, expression in mammalian cells is often required to ensure that PTMs are correctly added and the protein is correctly folded and active (see §[Sec sec6.6]6.6 for a discussion of *GnTI* and *lec1* glycosylation-deficient mammalian cells). Alternatively, insect cells such as *Spodoptera frugiperda* (*e.g.* Sf9 and Sf21) and *Trichoplusia ni* can be used (Altmann *et al.*, 1999 ▸; Jarvis, 2009 ▸).

## 5. Key techniques for determining protein stability   

The relative merits of the three main structural methods for assessing protein stability are shown in Table 2 ▸. Given the solid-state nature of protein crystallography it is often difficult to crystallize dynamic and disordered proteins, and for this reason NMR spectroscopy has been one of the main tools used to study IDPs such as p53 and CREB (Brutscher *et al.*, 2015 ▸; Dunker & Oldfield, 2015 ▸; Mujtaba *et al.*, 2004 ▸; Fig. 2 ▸ *d*). NMR is extremely useful for assessing both secondary and tertiary structure in dynamic and disordered systems (see §[Sec sec5.1]5.1). The higher resolution of crystal structures make them a particularly attractive method for determining changes at the primary-structure level. For example, uncharacterized PTMs, such as glycosylation, can often be interpreted directly from the electron-density maps if the experimental data are of sufficiently high resolution. Similarly, given sufficiently high-resolution maps, cryo-EM can be a powerful technique for determining protein stability and dynamics at the quaternary level (see §[Sec sec5.2]5.2).

### 5.1. Nuclear magnetic resonance (NMR)   

NMR spectroscopy is a powerful method for the determination of the stability of proteins in solution (Bieri *et al.*, 2011 ▸; Kwan *et al.*, 2011 ▸). The method is highly complementary to X-ray structure analysis, but given its ability to analyze structures in the solution state it is of tremendous value for assessing protein *conformational* stability (Krishnan & Rupp, 2012 ▸).

**Figure 3:**
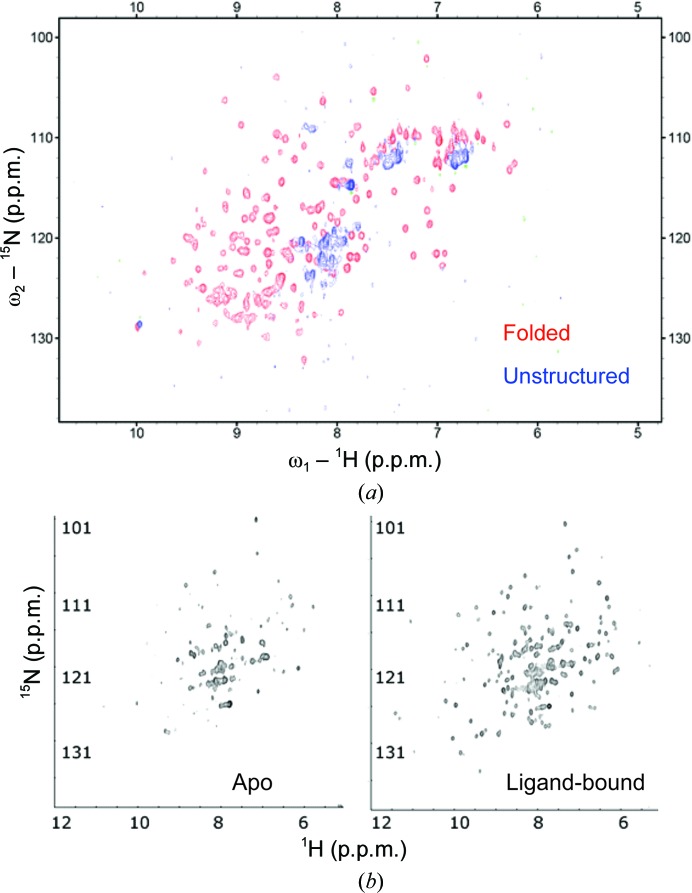
HSQC spectrum of folded, unstructured and apo and ligand-bound proteins. (*a*) Two-dimensional ^1^H–^15^N heteronuclear single-quantum coherence (HSQC) NMR spectrum showing the distinct discrimination in the region below 8.3 p.p.m. in ω_1_ identifying a folded protein (red, sharp peak contours) compared with the wide and unresolved peaks for disordered protein sample (blue contours). Image courtesy of Simon Colebrook, Department of Biochemistry, Oxford University and Joanne Nettleship, Oxford Protein Production facility. (*b*) HSQC spectrum of apo and ligand-bound protein. The two-dimensional ^1^H–^15^N HSQC NMR spectrum of bacterial methionine aminopeptidase (bMAP) with (right) and without (left) a tightly bound novel inhibitor (Evdokimov *et al.*, 2007 ▸). Note the drastic improvement in the discrimination of the spectrum for the bMAP–ligand complex compared with the apoprotein. The crystals of the bMAP–ligand complex diffracted to 0.9 Å resolution. Image courtesy of Artem Evdokimov, Procter & Gamble Pharmaceuticals, Mason, Ohio, USA. Figure adapted from Rupp (2015 ▸).

The fact that NMR can readily distinguish between folded and unfolded proteins, and detect the presence of disordered and unstructured regions, makes it inherently useful as a diagnostic tool for crystallization experiments (Fig. 3 ▸ *a*). Modern instruments can extract this information with minimal sample requirement (∼10 n*M*) and a simple one-dimensional proton NMR spectrum can provide information on the conformational stability of the macromolecule. Specifically, as a result of the principal inverse relation between spin–spin relaxation time and the peak width, large soluble aggregates will not yield an interpretable high-resolution NMR spectrum. For non-aggregated protein samples that yield usable one-dimensional NMR spectra, good discrimination in the backbone amide region below 8.3 p.p.m., as well as peaks at around ∼1 p.p.m., are indicative of folded protein (Rehm *et al.*, 2002 ▸). Furthermore, two-dimensional heteronuclear single-quantum coherence (HSQC) NMR spectra can be used to analyze the difference between folded and unstructured protein (Fig. 3 ▸ *a*) and also to compare apoprotein and ligand-bound complexes (Figs. 3 ▸ *b* and 3 ▸ *c*). Such a two-dimensional spectrum maps the backbone amide groups according to their ^1^H and ^15^N resonance frequencies. This method necessitates the production of ^15^N-labeled protein and requires larger amounts of sample compared with the more qualitative one-dimensional spectral analysis (Zhao *et al.*, 2004 ▸).

One of the main benefits of NMR methods is that the effect of environmental conditions, such as pH, temperature or ligand binding, can be readily varied and studied in a near-native solution state. Additionally, the nondestructive nature of NMR spectroscopy means that the samples can also be used for subsequent crystallization experiments, and high-throughput structure-determination facilities often combine NMR screening with crystallization experiments.

### 5.2. Electron microscopy   

The high conformational heterogeneity of proteins, especially of large multi-domain protein–protein complexes, can often hinder crystallization efforts. Mutational variants and different combinations of protein partners may need to be screened for suitability for crystallization. To facilitate this, electron microscopy (EM) can be used to directly visualize the sample and assess the level of heterogeneity. In the best-case scenario three-dimensional cryo-EM reconstructions can be generated, but this can be time-consuming and often requires substantial efforts in screening for suitable data-collection parameters. However, the generation of raw images of negatively stained particles is straightforward and requires very little protein (typically <10 µg). This method is already routinely practiced by EM practitioners to screen for good samples to move forward for cryo-EM analysis, but has recently been adapted for crystallography. However, the negative stain (*e.g.* uranyl formate) may introduce artifacts or otherwise disrupt the protein sample. Additionally, the protein (or protein–protein complex) needs to be relatively large (>150 kDa) in order to be imaged. If these caveats can be overcome then simple negative staining can be a powerful technique for providing a low-resolution glimpse of the protein sample especially for rapid large-scale screening purposes. Furthermore, simple image analysis of the particles can be used to produce a two-dimensional class average and quantify the conformational classes within the protein sample. Thus, EM can guide the screening of protein constructs for further structural studies, such as crystallization or full three-dimensional cryo-EM reconstructions (Pugach *et al.*, 2015 ▸; Julien *et al.*, 2015 ▸). It is important to note that protein crystallography generally works well with protein complexes of <150 kDa, which makes these two techniques highly complementary.

With technological innovations pushing its data sets beyond 3 Å resolution, cryo-EM techniques are becoming an increasingly useful tool for screening of both protein *conformational* stability and *compositional* stability. Examples of recent studies using EM to assess protein stability and dynamics include the structural transitions of αβ-tubulin (Alushin *et al.*, 2014 ▸) and the stability of the HBV capsid protein (Selzer *et al.*, 2015 ▸).

### 5.3. Spectroscopic methods   

Spectroscopic methods are primarily used to assess the stability of the protein at the level of secondary structure. Secondary structure can be analyzed using a variety of spectroscopic methods including circular dichroism (CD), Fourier transform infrared (FT-IR) and Raman spectroscopy (Pelton & McLean, 2000 ▸). CD spectroscopy, or spectropolarimetry, of proteins is carried out in the far-ultraviolet range (170–250 nm) and has seen a recent resurgence in usage as a result of the development of synchrotron-radiation circular dichroism (SRCD), which can operate at lower wavelengths (Whitmore & Wallace, 2008 ▸; Wallace & Janes, 2010 ▸). SRCD data are now publicly available in a central repository at the Protein Circular Dichroism Data Bank (PCDDB), which currently contains 529 entries (Whitmore *et al.*, 2011 ▸; http://bit.ly/1OrNzrP). Using this technique, a twin minimum in the ellipticity spectrum at 208 and 222 nm is produced by α-helical content, whereas a single minimum at 204 or 217 nm is suggestive of random-coil or β-sheet content, respectively. Example uses of UV-CD for assessing protein stability include a study of the plant membrane protein MBP-b_6_. The percentages of helix, sheet, turn and unordered secondary structure were determined and it was demonstrated that *n*-dodecyl-β-d-maltoside and Triton X-100 both preserved the correct secondary structure, whereas sodium dodecyl sulfate was shown to disrupt the secondary structure (Surma *et al.*, 2014 ▸).

Infrared spectroscopy can also be used to identify secondary-structure content (Barth, 2007 ▸). This technique is used to analyze changes in the bond oscillation of amide groups which result from differences in the hydrogen-bonding pattern. Refinements of this technique include femtosecond two-dimensional infrared spectroscopy (2D-IR), which is more sensitive in detecting structural differences (Demirdöven *et al.*, 2004 ▸).

### 5.4. Melting-temperature analysis   

Assessment of protein stability, particularly thermodynamic stability, is of tremendous value for the crystallization of both soluble and membrane proteins. The global thermal stability of a protein can be represented by its thermal denaturation midpoint or melting temperature (*T*_m_). Methods used for the determination of *T*_m_ include Thermofluor (also known as differential scanning fluorimetry; DSF; Boivin *et al.*, 2013 ▸; Ericsson *et al.*, 2006 ▸; Reinhard *et al.*, 2013 ▸) and differential scanning calorimetry (DSC; Sanchez-Ruiz, 1995 ▸; Privalov & Dragan, 2007 ▸; Bruylants *et al.*, 2005 ▸).

The *T*_m_ is often determined using the Thermofluor technique using a reporter dye such as SYPRO Orange or 1-anilinonaphthalene-8-sulfonate (ANS). The reporter dye undergoes a change in fluorescence properties upon binding to the hydrophobic core of the unfolded protein (Semisotnov *et al.*, 1991 ▸). A major advantage of the technique is that very little protein is required (typically <<1 mg) and the only equipment required is a readily available qPCR machine. One interesting application of this technique is the detection of ligand binding to proteins of unknown function. For example, by screening a library of 3000 compounds this technique was used to determine that an essential gene from *Streptococcus pneumoniae* was a nucleoside diphospho-keto-sugar aminotransferase (Carver *et al.*, 2005 ▸). Additionally, Thermofluor techniques can be used for the quantitation of protein–protein interactions. This has been used to analyze the stabilizing effect of maltose-binding protein on ankyrin-repeat proteins *via* the production of a series of alanine mutants to probe the interaction (Layton & Hellinga, 2011 ▸). Using such techniques, it is possible to screen hundreds of protein truncations (see §[Sec sec6.1]6.1) and buffer conditions (see §[Sec sec6.2]6.2) on a high-throughput scale (Boivin *et al.*, 2013 ▸; Ristic *et al.*, 2015 ▸; Seabrook & Newman, 2013 ▸; Reinhard *et al.*, 2013 ▸). Melting-temperature methods are also applicable for the assessment of the thermo­dynamic stability of membrane proteins in detergents and have been successfully applied to the analysis of the stability of the acetylcholine receptor in Brij-35 (Yeh *et al.*, 2006 ▸).

Potential caveats of the Thermofluor technique include the possibility of protein–dye interactions that many adversely affect the protein stability, a phenomenon that was observed in the study of GroEL when using the ANS dye (Smoot *et al.*, 2001 ▸). Additionally, aberrant or false-positive thermal shifts are fairly common, and careful analysis of the melt-curve data must be carried out; tools such as *Meltdown* are available to assist in this effort (Rosa *et al.*, 2015 ▸). It is also important to note that not all proteins unfold in a well-defined sigmoid melting curve, including proteins with high disulfide-bond content (*e.g.* albumin) and proteins from thermophilic organisms such as *T. maritima*. Finally, a negative Thermofluor result (*i.e.* no change in *T*_m_) does not necessarily indicate a lack of binding for small molecules, but could simply mean that the small molecule does not stabilize the protein further. This is of particular relevance for proteins which have high melting temperatures in the absence of ligands.

## 6. Improving protein stability and the concept of crystallizability   

Many techniques are available for increasing the crystallizability of a protein, and the central theme of these techniques is the improvement of both the compositional and the conformational stability of the protein sample (Table 4 ▸). These methods include truncations, buffers, ligands, purification tags, reductive methylation, surface-entropy reduction (SER), *in situ* proteolysis, Thermofluor, deuterium-exchange mass spectrometry (DXMS) and disulfide engineering. A complementary review of protein-engineering approaches for improving the properties of proteins for crystallization studies is provided by Ruggiero *et al.* (2012 ▸).

Several software packages and algorithms have been developed to assess the so-called crystallizability of a protein (Smialowski & Frishman, 2010 ▸; Derewenda, 2010 ▸; Ruggiero *et al.*, 2012 ▸). These include *DisMeta* (Huang *et al.*, 2014 ▸), *XtalPred* (Jahandideh *et al.*, 2014 ▸; Slabinski *et al.*, 2007 ▸), *POODLE* (Shimizu, 2014 ▸), *MFDp*2 (Mizianty *et al.*, 2014 ▸), *MoRFpred* (Disfani *et al.*, 2012 ▸), *RFCRYS* (Jahandideh & Mahdavi, 2012 ▸), *XANNpred* (Overton *et al.*, 2011 ▸), *SVMCRYS* (Kandaswamy *et al.*, 2010 ▸), *SCMCRYS* (Charoenkwan *et al.*, 2013 ▸), *CRYSTALP*2 (Kurgan *et al.*, 2009 ▸), *MetaPPCP* (Mizianty & Kurgan, 2009 ▸) and *ParCrys* (Overton *et al.*, 2008 ▸). Many of these packages use a template-based approach to analyze the propensity of a protein to crystallize by comparison with known crystal structures. However, some of these packages, including *XtalPred*, *POODLE* and *DisMeta*, utilize sequence-based predictions to identify regions of low complexity, disorder, transmembrane and signal peptides.

It is important to note that the propensity for disorder calculated by most of these methods, and in turn the propensity for crystallization, is largely predicted on the basis of a single polypeptide chain in isolation. Clearly, protein–protein complexes can often result in stabilization of the constituent proteins, as is commonly observed for IDPs (see §[Sec sec2.2]2.2). Therefore, particularly for crystallographic studies, it is often essential to study the protein–protein complex as a whole; the individual proteins are often too disordered or unstable when uncomplexed.

### 6.1. Truncations and domain selection   

Selection of the shortest possible domain is often preferable and computational tools such as Expression of Soluble Proteins by Random Incremental Truncation (ESPRIT) and combinatorial domain hunting (CDH) are available to assist in this effort (Reich *et al.*, 2006 ▸; Yumerefendi *et al.*, 2010 ▸). Truncation of a protein to the shortest possible fragment can often be a key factor in successful structure solution. For example, amyloid fibers are of tremendous medical importance in diseases such Alzheimer’s and prion diseases and their partially disordered structure has traditionally hindered structural analysis using crystallographic techniques. However, shorter fragments of only 6–7 amino acids in length, which also form fibrils, were used to produce microcrystals and to determine the structure (Moshe *et al.*, 2016 ▸; Sawaya *et al.*, 2007 ▸). Another example is the production of structured truncation arrays of a target protein generated using polymerase incomplete primer extension cloning methods (Klock & Lesley, 2009 ▸). Using this technique, structural genomics consortia such as the Joint Center for Structural Genomics (JCSG) and the Structural Genomics Institute, Karolinska Institutet have been able to generate several thousands of truncations for targets recalcitrant to crystallization (Klock *et al.*, 2008 ▸; Gräslund *et al.*, 2008 ▸).

In addition to truncations of the protein, it is also important to consider other mutations of the protein. Stability-enhancing mutations in membrane proteins are surprisingly common and some estimates suggest that ∼10% of random mutations will confer some level of stability on the protein (Bowie, 2001 ▸). For example, two valine-to-alanine substitutions in the transmembrane portion of the M13 coat protein were found to enhance thermal stability (Deber *et al.*, 1993 ▸). The reasons for the stability-enhancing effects of mutations are not always immediately obvious from analysis of the structure. It has been suggested that membrane proteins are required to be inherently flexible, and therefore conformationally unstable, in order to maintain function in the restricted environment of the membrane (Bowie, 2001 ▸).

### 6.2. Buffer screening   

The buffer in which a protein is solubilized exerts an influence on its stability (Davis-Searles *et al.*, 2001 ▸). Therefore, buffer screening is a powerful method for stabilizing proteins for crystallographic applications and also for the formulation of biologics. One of the primary methods used for high-throughput buffer screening is Thermofluor (see §[Sec sec5.4]5.4). Using such approaches it is possible to screen libraries of hundreds of different buffers and pH combinations, and the stabilizing effect can be easily inferred from the change in *T*_m_ (Δ*T*_m_; Reinhard *et al.*, 2013 ▸; Ristic *et al.*, 2015 ▸). Using these approaches, interesting protein-stabilizing buffers have been identified. For example, citrate, bis-tris and *N*-(2-acetamido)iminodiacetic acid (ADA) have all been identified as having statistically significant stabilizing effects on the proteins tested (Ristic *et al.*, 2015 ▸). As a more extreme example of buffer screening, IIPs (see §[Sec sec2.2]2.2) such as V22-SH3 are insoluble in traditional buffer systems and can only be solubilized in pure water (Liu & Song, 2009 ▸).

### 6.3. Ligands and additive screening   

Ligand binding can also significantly help to stabilize the protein, particularly from the perspective of *conformational* stability. Co-crystal structures of proteins bound to cofactors, prosthetic groups, substrates, drugs and inhibitors are often the holy grail of structural biology; somewhat fortunately for the structural biologist, ligands often have a stabilizing effect on the protein and can increase the chances of successful crystallization. It is important to remember that although soaking of compounds through the crystal lattice to the active site is often possible, it may also bring about conformational changes in the protein on binding of the ligand. Therefore, *ab initio* crystal screening in the presence of the ligand may be required in order to obtain crystals (Hassell *et al.*, 2007 ▸). The judicial use of bioanalytical techniques, such as Thermofluor and NMR, is key for guiding the successful production of a ligand-bound crystal structure (see §[Sec sec5]5).

In addition to the stabilizing effects of small-molecule ligands, it is also possible to identify ions and other organic additives that stabilize the protein or even the crystal. For example, Thermofluor was used to identify magnesium ions as a stabilizing influence on the enzyme DapD from *Mycobacterium tuberculosis*, and the subsequent addition of MgCl_2_ to the crystallization solution resulted in larger crystals (Reinhard *et al.*, 2013 ▸; Schuldt *et al.*, 2009 ▸). In this case, stabilization of the quaternary structure results from the addition of magnesium ions, and two tightly coordinated Mg^2+^ ions were identified in the homotrimer interface of the crystal structure (Schuldt *et al.*, 2009 ▸). Other examples of stabilizing additives include the commonly used precipitants polyethylene glycol (PEG) and 2-methyl-2,4-pentanediol (MPD), which are both often observed bound to crystal structures. In the case of MPD it has been proposed that it stabilizes the protein by promoting the hydration of the protein surface by binding to exposed hydrophobic surface residues such as leucine (Anand *et al.*, 2002 ▸). Building on this theme, additive screens such as ‘Silver Bullets’ (Hampton Research) have been assembled that can stabilize or initiate cross-linking between proteins further promoting crystal lattice formation (McPherson & Cudney, 2006 ▸).

### 6.4. Fused affinity tags and crystallization chaperones   

As discussed in §[Sec sec4.1]4.1, affinity purification tags such as MBP are often used to aid in both protein stability and solubility during the course of protein expression and purification. Larger tags such as MBP are usually removed prior to crystallization trials, as the flexibility of the tag can interfere with crystal packing. However, in some cases smaller tags such as His can often be left on without unduly affecting crystal packing or protein function (Bucher *et al.*, 2002 ▸). The primary reason for removing large tags is the reduced conformational stability resulting from the flexible linker between the target protein and its affinity tag. Several groups have successfully ‘engineered out’ this linker flexibility by inserting a string of alanine residues in place of the usual protease cleavage site in the linker (Smyth *et al.*, 2003 ▸). This concept has resulting in several crystal structures of proteins fused to MBP, including gp21 (Kobe *et al.*, 1999 ▸), SarR (Liu *et al.*, 2001 ▸), MATa1 (Ke & Wolberger, 2003 ▸) and MCL1 (Clifton *et al.*, 2015 ▸). This concept has led to the idea of crystallization chaperones (Bukowska & Grütter, 2013 ▸). Example uses of crystallization chaperones include the application of Fab antibody fragments to study the neurotransmitter sodium symporter LeuT in various conformational states (Krishnamurthy & Gouaux, 2012 ▸) and the fusion of T4 lysozyme to G-protein-coupled receptor (Zou *et al.*, 2012 ▸). These approaches are proving to be useful for the stabilization of membrane proteins and as aids in their structure determination (Lieberman *et al.*, 2011 ▸).

### 6.5. Reductive alkylation   

Modification of the protein surface is a well established strategy for enhancing protein crystallization and can be achieved using site-directed mutagenesis (see §[Sec sec6.6]6.6) or chemical modification (Derewenda, 2004 ▸). A common method of chemical modification is the reductive methylation of the ∊-amino groups of solvent-exposed lysine residues. This is performed using the reducing agents dimethylamine–borane and formaldehyde (Means, 1977 ▸). In recent years, this technique has become one of the workhorse ‘salvage’ techniques of structural genomics consortia (Tan *et al.*, 2014 ▸; Walter *et al.*, 2006 ▸; Sledz *et al.*, 2010 ▸). Reductive methylation is believed to function *via* the introduction of new surface contacts, therefore promoting crystal lattice formation (Sledz *et al.*, 2010 ▸).

Recent developments of the technique include the use of ethylation and isopropylation, although fewer targets are available to assess the performance of such techniques (Tan *et al.*, 2014 ▸). Another important development of the method is the use of cysteine alkylation for the structure determination of membrane proteins. This method was first used for the determination of the β_1_-adrenergic G-protein-coupled receptor structure (Warne *et al.*, 2008 ▸) and is in common use for many GPCR studies (Columbus, 2015 ▸). Cysteine alkylation stabilizes the protein by preventing the formation of disulfide bonds, and in the case of the β_1_-adrenergic receptor functions by stabilizing the monomers and preventing oligomers forming (Mathiasen *et al.*, 2014 ▸).

In addition to alkylation, other chemical modifications such as fluorination can be carried out. Fluorine is all but absent from biological systems, but stabilizes proteins as a result of the ‘fluorous effect’ (Buer & Marsh, 2014 ▸; Marsh, 2014 ▸). This effect results in an unusual propensity to undergo phase separation and causes an increase in the buried surface area in the hydrophobic core of fluorinated proteins. Fluorinated proteins can be generated using the highly fluorinated amino acid hexafluoroleucine. The crystal structure of a designed four-helical bundle protein, α4H, reveals that the fluorinated residues pack well into the hydrophobic core of the protein with little perturbation of the structure (Buer *et al.*, 2012 ▸). This method would clearly perturb the structure and the function of some proteins, but may be a method worthy of further investigation for enabling structural studies of very unstable proteins.

### 6.6. Surface mutagenesis, surface-entropy reduction (SER) and deglycosylation   

Mutagenesis of surface-exposed amino acids is a proven method for engineering proteins with improved stability and chance of crystallization (Derewenda, 2004 ▸, 2010 ▸; Derewenda & Vekilov, 2006 ▸). Amongst the first uses of mutagenesis to enhance crystallizability was the transplant of key crystal contacts from the rat ferritin structure onto the human ortholog, which was previously unsolved (Lawson *et al.*, 1991 ▸). In this example, Lys86 of the human protein was mutated to Glu to mimic the Ca^2+^-binding site observed in the crystal contacts of the rat ortholog. Clearly, this technique is useful if an orthologous structure is available. However, this is often not the case and one is shooting in the dark when choosing surface residues to mutate.

To address this issue, Derewenda and coworkers made a series of mutations to RhoGDI targeting glutamate and lysine residues on the surface of the protein (Longenecker *et al.*, 2001 ▸; Mateja *et al.*, 2002 ▸; Czepas *et al.*, 2004 ▸). These residues have high conformational entropy and rarely participate in protein–protein interfaces within the crystal (Lo Conte *et al.*, 1999 ▸; Baud & Karlin, 1999 ▸). Therefore, they represent attractive targets for modification of crystal contacts. In an attempt to reduce the conformational entropy on the protein surface, these residues were mutated to either alanine, arginine or aspartate. The original set of mutable residues has more recently been expanded to include glutamine. The *SERp* server is an excellent resource for identifying such residues (Goldschmidt *et al.*, 2007 ▸, 2014 ▸; http://bit.ly/1LFjdyk). This site evaluates the solvent exposure, secondary structure, surface entropy and evolutionary conservation for sets of glutamate, glutamine or lysine residues. Analysis of the evolutionary conservation is used as a guide to avoid the mutation of residues that may be structurally or functionally significant. Finally, the site suggests appropriate clusters of residues matching these criteria as suitable for mutagenesis. These techniques have been used in conjunction with the fused affinity-tag and molecular-chaperone approach discussed above (see §[Sec sec6.4]6.4) to determine the structure of a RACK1A-MBP fusion protein (Moon *et al.*, 2010 ▸).

Finally, the presence of PTMs (see §[Sec sec3.1]3.1) on the surface of the protein, in particular glycosylation, must be carefully considered. N- and O-linked sugars occur frequently on the surface of eukaryotic proteins and their chemical heterogeneity and conformational freedom can result in significant conformational variability. Strategies to deal with glycosylation on the protein surface include expression using a deglycosylation-deficient CHO cell strains such as *lec1* (Puthalakath *et al.*, 1996 ▸) or an HEK293S strain such as *GnTI* (Reeves *et al.*, 2002 ▸). Another strategy is the mutagenesis of consensus glycosylation sites, such as the Asn-*X*-Ser/Thr sequon, which is targeted for N-linked glycosylation (Mellquist *et al.*, 1998 ▸), and Ser or Thr residues, which are targeted for O-linked glycosyl­ation. Alternatively, endoglycosylases such as endoglycosidase H (Endo H) and PNGase F can be used. In the case of PNGase F the glycosylated asparagine residue is converted to an aspartic acid, thus removing the sugar completely. Another approach is the use of N-glycosylation inhibitors such as swainsonine and kifunensine (Elbein, 1987 ▸). An effective combination approach uses glycosylation inhibitors during expression, followed by treatment with Endo H. Such an approach has been used to generate diffraction-quality crystals of sRPTPμ and s19A (Chang *et al.*, 2007 ▸). Also, prudent selection of the expression system can be deployed to vary the glycosylation patterns. For example, Sf insect cells generally produce simpler glycosylation patterns of the GlucNAcMan_5_ type and often glycosylate at fewer sites, whereas yeast cells can often hyperglycosylate proteins.

It must be noted that the presence of glycans on the protein surface can sometimes aid in crystallization by mediating important crystal contacts. For example, a complex of the densely glycosylated *Hepatitis C virus* E2 (HCV E2) glycoprotein bound to a broadly neutralizing antibody failed to crystallize using numerous deglycosylation strategies, including Endo H treatment or N-linked glycan-sequon mutagenesis. In this example, only the fully glycosylated HCV E2 protein crystallized, and the resulting structure revealed a key crystal contact mediated by an N-linked glycan interacting non­specifically with a neighboring symmetry mate within the crystal (Kong *et al.*, 2013 ▸). Full or partial glycosylation may also be necessary to crystallize complexes involving glycan-dependent protein interactions (Kong *et al.*, 2014 ▸). These examples illustrate that glycosylation may also have a stabilizing effect on proteins, especially for crystallization, and the presence of glycans is not always deleterious.

### 6.7. *In situ* proteolysis   

The presence of proteases in a protein sample submitted for crystallization trials is clearly of significance and can lead to significant composition instability over the time course of the experiment. For example, it was serendipitously discovered that a penicillium fungus growing in a crystallization drop was responsible for cleaving ∼200 residues off the yeast CPSF-100 protein and was essential for successful crystallization (Mandel *et al.*, 2006 ▸; Bai *et al.*, 2007 ▸). This finding initiated so-called *in situ* proteolysis, in which protein samples are crystallized in the presence of a panel of proteases such as trypsin and chymotrypsin (Dong *et al.*, 2007 ▸; Wernimont & Edwards, 2009 ▸). More recently, these techniques have been combined with mass-spectrometric analysis to help identify peptide fragments that are stable over the time frame of a crystallization experiment (Gheyi *et al.*, 2010 ▸). In a similar fashion, limited proteolysis can be used to identify stable domains of membrane and globular proteins. The membrane protects the transmembrane portions of the protein from protease cleavage and more compact forms of the membrane protein can be produced. Such an approach was used to generate a stable 55 kDa construct of the P pilus PapC that was subsequently used for crystal structure determination (Remaut *et al.*, 2008 ▸).

### 6.8. Thermal stability screening   

**Figure 4:**
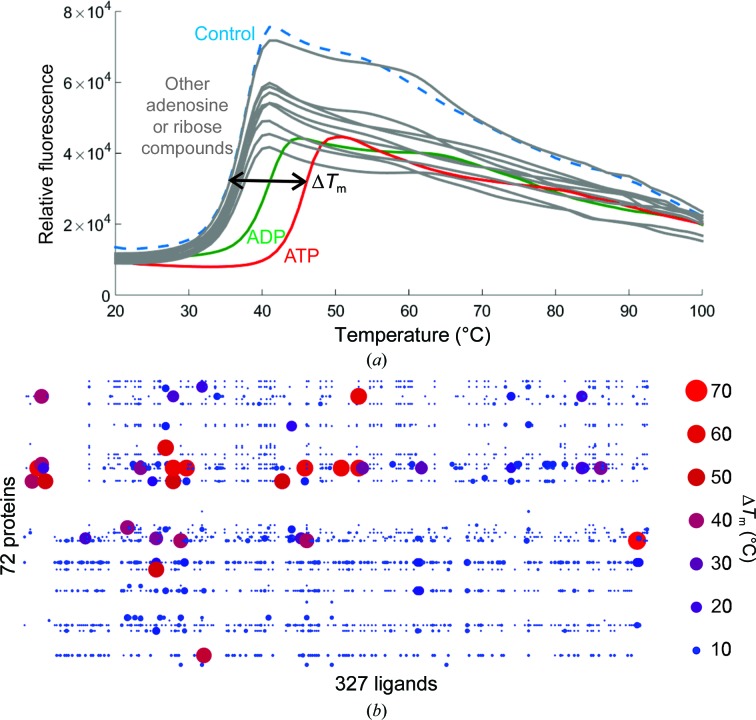
Thermofluor assay of protein melting temperature in the presence of stabilizing ligands. (*a*) Example melting curves for a protein of unknown function from *Eubacterium siraeum* (ZP_02421384.1) in the presence of various adenosine- and ribose-containing ligands (grey), adenosine diphosphate (ADP, green), adenosine triphosphate (ATP, red) and control sample with no ligand (blue dashed line). ADP and ATP result in a shift in melting temperature (Δ*T*_m_) of 3 and 8°C, respectively. (*b*) Matrix of 72 proteins of unknown function screened in a Thermofluor assay against a panel of 327 ligands. The Δ*T*_m_ is indicated by the size and color of the data points ranging from 10 to 70°C. Data kindly provided by Anna Grezchnik of the Joint Center for Structural Genomics (JCSG).

The thermal stability of a protein displays a good level of correlation with the crystallizability of a protein. For example, a large-scale Thermofluor study of 657 protein samples showed that only 23% of the proteins with a *T*_m_ of <43°C yielded crystals. In contrast, 49% of the proteins with a *T*_m_ of >45°C yielded crystals (Dupeux *et al.*, 2011 ▸; see §[Sec sec6.4]5.4 and Fig. 4 ▸). However, beyond 45°C the *T*_m_ does not appear to be particularly predictive of crystallizability; there is no significant increase in crystallization frequency for proteins with a *T*_m_ of between 45 and 96°C (Dupeux *et al.*, 2011 ▸). Furthermore, there are no known structural features that correlate well with *T*_m_ (Kumar *et al.*, 2000 ▸), and the *T*_m_ appears to be highly solvent-dependent (Faria *et al.*, 2004 ▸). It is important to note that other features of the melting curve, aside from the *T*_m_, may be useful to the crystallographer. For example, a high initial signal generated by a Thermofluor assay may indicate that an exposed hydrophobic surface of the native protein may be interacting with the dye used in the assay. Proteins displaying such profiles appear to have a lower rate of crystallization (36.6%; Dupeux *et al.*, 2011 ▸). Similarly, a wide thermal denaturation peak can be indicative of noncooperative unfolding behavior, which might be a useful consideration for construct modification (Morar-Mitrica *et al.*, 2013 ▸). Ultimately, measures of thermal stability may be useful as a broad, qualitative assessment of the suitability of a protein for crystallization.

### 6.9. Deuterium-exchange mass spectrometry (DXMS)   

**Figure 5:**
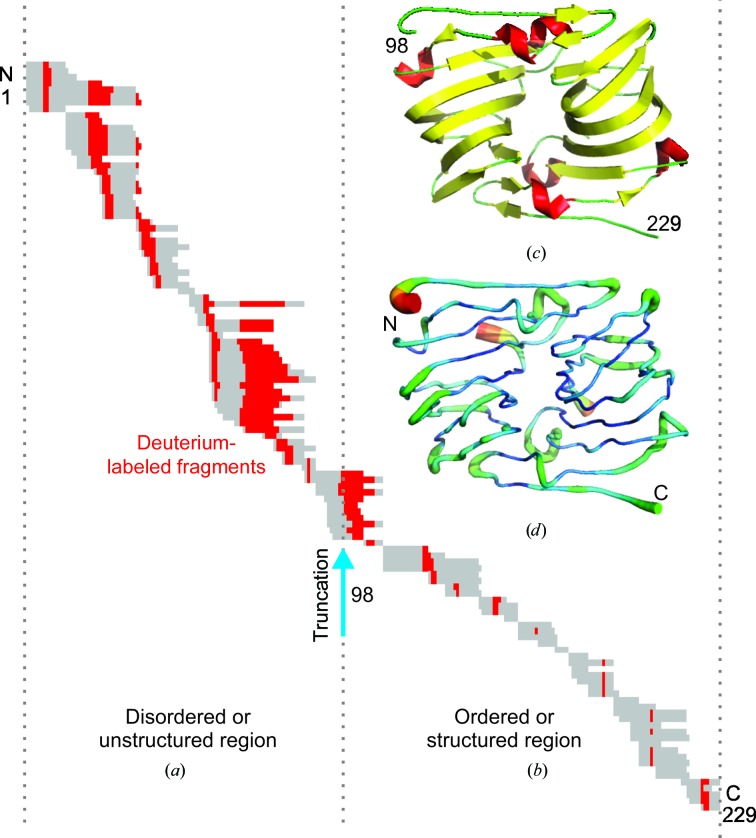
Deuterium-exchange mass spectrometry (DXMS) analysis. DXMS was used to guide the construct design used for determining the crystal structure of a putative ethanolamine-utilization protein from *Salmonella typhimurium*. (*a*) The left side of the figure shows that the N-terminal portion of the protein is more disordered, or unstructured in solution, as the backbone amide protons are more susceptible to exchange. Deuterium-labelled proteolytic fragments are highlighted in red. (*b*) The right side of the figure shows that the C-terminal portion of the protein is more ordered, or structured in solution, as the backbone-amide protons are less susceptible to exchange. The region selected for truncation is denoted by a blue arrow. (*c*) Ribbon diagram of the final crystal structure determined for residues 98–229 showing the compact and ordered structure (loops are shown in green, α-helices in red and β-­strands in yellow; PDB entry 2pyt; Joint Center for Structural Genomics, unpublished work). (*d*) The same structure colored according to the *B*-factor value, highlighting the stable core of the protein in blue and the more flexible outer regions in green through red. Data kindly provided by Scott Lesley of the Joint Center for Structural Genomics (JCSG).

Prior to crystallization, it is often desirable to remove protein termini and low-complexity regions owing to their inherent flexibility (Derewenda, 2010 ▸). Disordered regions of the protein often hamper crystallization and the design of protein constructs trimmed of such regions can be guided using deuterium-exchange mass spectrometry (DXMS; Englander, 2006 ▸; Konermann *et al.*, 2011 ▸; Spraggon *et al.*, 2004 ▸; Figs. 5 ▸ *a* and 5 ▸ *b*).

Using this technique, proteins are exposed to deuterated solvent and the deuterium is allowed to exchange with the backbone amide protons of the protein. The exchange rates are dependent on the degree of solvent exposure and the integrity of the local secondary structure. Therefore, this method gives a measure of the conformational flexibility of the protein in solution (Englander & Kallenbach, 1983 ▸). These rates can be obtained by measuring the change in the mass of the deuterated peptides using proteolytic mass spectrometry. DXMS has been successfully applied to structural genomics targets, guiding the deletion of flexible regions that previously hindered the growth of diffraction-quality crystals (Spraggon *et al.*, 2004 ▸; Pantazatos *et al.*, 2004 ▸; Fig. 5). Although the utility of DXMS is often limited by the lengths of the proteolytic fragments that can be produced, sometimes restricting the area of investigation to less than 50% of the protein sequence, it can often provide a key insight for the design of crystallizable constructs. Furthermore, when coupled with structure, DXMS can be mapped onto the protein structure to provide a three-dimensional map of flexibility that can further guide protein-engineering efforts and mechanistic studies (Guttman *et al.*, 2012 ▸; Kong *et al.*, 2010 ▸; Zhang *et al.*, 2012 ▸; Chung *et al.*, 2011 ▸).

### 6.10. Disulfide engineering   

Disulfide engineering is a well established method for stabilizing proteins and for studying and modifying protein function and dynamics (Dombkowski *et al.*, 2014 ▸). Intermolecular disulfide bonds between proteins in the crystal lattice have been observed and have led to the coining of the phrase ‘spontaneously polymerizing protein crystals’ (Quistgaard, 2014 ▸). Disulfide engineering can be used to introduce these intermolecular disulfides into the protein in an attempt to stabilize the lattice and promote crystallization. Furthermore, studies have shown that symmetrical proteins, such as homodimers, tend to crystallize more readily (Wukovitz & Yeates, 1995 ▸). Using T4 lysozyme as a model system, it has been demonstrated that the introduction of disulfide bonds can be used to make monomeric proteins dimerize and increasing the chance of lattice formation (Banatao *et al.*, 2006 ▸; Heinz & Matthews, 1994 ▸). This has been termed ‘synthetic symmetrization’ and can be a useful tool for assisting in the crystallization of monomeric proteins and protein–protein complexes which display asymmetry. Proteins that have been successfully crystallized using this approach include CelA from *Thermotoga maritima* (Forse *et al.*, 2011 ▸). Tools for identifying residues suitable for disulfide engineering are available, including *Disulfide by Design* 2.0 (Craig & Dombkowski, 2013 ▸; http://bit.ly/1NbV2tQ). One caveat of this approach is the potential of disulfide bonds to adopt different conformations that may promote conformational flexibility. For example, the disulfide bond connecting the I-EGF1 and I-EGF2 domains of β_2_ integrin is able to accommodate a >20 Å hinge motion between the domains (Shi *et al.*, 2007 ▸; Smagghe *et al.*, 2010 ▸).

## 7. Future outlook   

Protein stability does mean many different things to many different scientists. However, on a global level, it can be considered as the ability of a protein to maintain structure and function in a particular environment. If the environment of interest is normal physiological conditions then the net summation of all contributing forces must add up to provide a small negative Δ*G*, therefore favoring a stable folded protein. However, not all proteins operate in standard physiological environments and many other factors must be considered. For example, many proteins, such as FeFe hydrogenase, are sensitive to oxygen and changes to the structure must be considered under an oxygen-free environment (Mulder *et al.*, 2011 ▸). However, the growth of such enzymes under anaerobic conditions is both costly and difficult to achieve on a large scale, and considerable effort is being made to generate oxygen-tolerant hydrogenases for use on an industrial scale (Fritsch *et al.*, 2013 ▸). The stability of such enzymes is of great interest to the biofuels industry, with the potential for biological hydrogen-gas production (Kim & Kim, 2011 ▸). Similarly, light-sensitive proteins such as opsins, photolyase and photosystems I and II all have unique structural features that enable them to utilize the energy of photons to carry out biological functions. Other interesting examples include the light-, oxygen- and voltage-sensitive domains (LOVs) found in plants and algae that undergo conformational changes and covalent binding of an FMN moiety under illumination with blue light (Briggs, 2007 ▸; Kottke *et al.*, 2006 ▸). These examples illustrate how nonstandard environments must be considered in any discussion of protein stability.

Traditionally, discussions of protein stability have focused on soluble, folded and more classical globular proteins. However, large percentages of the proteome are predicted to contain unstructured, insoluble and aggregated proteins in the form of IDPs, IIPs and APRs (see §[Sec sec2.2]2.2). Such proteins, and their unwieldy conformational stability, represent a challenge for protein crystallographers, who usually simply remove such regions during the cloning stage. However, these families of proteins are of tremendous medical importance and deletion of these regions at the cloning stage is no longer meaningful. Crystallographic techniques are evolving to help to deal with such proteins, particularly in the areas of microcrystallo­graphy, next-generation synchrotron sources and X-ray free-electron lasers (XFELs) (Neutze & Moffat, 2012 ▸; Spence *et al.*, 2012 ▸; Gruner & Lattman, 2015 ▸; Weckert, 2015 ▸; Moukhametzianov *et al.*, 2008 ▸; Igarashi *et al.*, 2008 ▸; Fischetti *et al.*, 2009 ▸).

Recent developments in XFEL light sources are enabling higher resolution studies of protein dynamics on a femto­second timescale (Uervirojnangkoorn *et al.*, 2015 ▸). From a crystallization perspective, such light sources present a unique set of challenges to the crystallographer that are somewhat orthogonal to the traditional problems faced when using more traditional diffraction methods. One such requirement is the need for microcrystalline sample material that can be injected into the laser path. Additionally, such techniques also allow larger and more dynamic protein structures to be determined, particularly those which possess conformational disorder and thus hinder the formation of larger, more ordered crystals. Recent examples using XFEL techniques include structures of the complex between synaptotagmin 1 and the neuronal SNARE (Zhou *et al.*, 2015 ▸). Clearly, XFEL technology, especially when coupled with associated hybrid methods, such as cryo-EM, ultrafast electron diffraction (UED) and double electron–electron resonance (DEER), will help mitigate many of the problems associated with protein conformation stability and its effect on protein crystallization (Wakatsuki, 2016 ▸). Next-generation synchrotron sources will be capable of providing smaller and brighter X-ray beams, and most importantly with a higher coherence fraction (Weckert, 2015 ▸). Such coherent beams will provide exciting opportunities for the study of protein dynamics on ever smaller timescales and will be essential for the study of unstable proteins that are currently inaccessible using traditional crystallographic techniques.

In summary, we will end this review with the title of an excellent paper by the late Fred Richards: *Protein stability: still an unsolved problem* (Richards, 1997 ▸). Although the problem is still largely unsolved, considerable progress has been made towards the study of protein stability, disorder and dynamics. Structural methods such as crystallography, NMR and cryo-EM are central to this endeavor and the exploration of innovative hybrid methods will be vital.

## Figures and Tables

**Table 1:** *Compositional* stability *versus conformational* stability: some important questions to ask when embarking on the crystallization of a protein and some factors to investigate for problem proteins

Global types of protein stability	Key questions to ask when trying to crystallize a protein	Required answers for successful crystallization	Factors to investigate if the answer is ‘No’
*Compositional* stability	Is the chemical makeup of the protein well defined?	Yes	Check amino-acid sequence. Check for PTMs, especially proteolysis. Purify protein more. Carry out more rigorous bioanalytical methods such as mass spectrometry and light scattering.
	Does the protein have a high level of chemical homogeneity?	Yes	
	Is the protein chemically stable in the crystallization conditions?	Yes	Use customized or less harsh crystallization screens. Explore different temperatures for screening.
	Is the protein stable over the course of the crystallization experiment?	Yes	
*Conformational* stability	Are there minimal disordered regions in the protein?	Yes	Redesign expression constructs to engineer out disordered or dynamic regions. Identify stabilizing protein partners or ligands.
	Does the protein have a minimal content of domains or regions that undergo dynamic variability over time?	Yes	

**Table 2:** Common measures of protein stability Definitions of protein stability at each structural level are shown along with common methods used to analyze the degree of stability. Asterisks denote the relative merits of the three main structure-determination techniques, with five asterisks denoting the optimal method. For example, NMR solution methods are often more favorable for studying dynamic processes and quaternary states as they are not influenced by crystal packing.

				Relative merits of structural methods		
Structural level	Definition of stability	Example biochemical processes or features	Common methods	Crystallography	NMR	EM
Primary	Change of amino-acid sequence or modification of amino acids	PTM Proteolysis Protein splicing	Half-life analysis SDS–PAGE Mass spectrometry Eastern and Western blotting	*****	****	*
Secondary	Change of α-helix, β-sheet and loop content	Secondary-structure formation Racemization Aromatic side-chain interactions Ligands	Circular dichroism (CD) Synchrotron-radiation CD UV-CD FT-IR 2D-IR Deuterium-exchange mass spectromety (DXMS)	*****	*****	*
Tertiary	Change of overall fold or protein conformation	Hydrogen bonding Hydrophobic interactions Conformational change Disulfide bonding Topology	ITC DSC Thermofluor	****	****	**
Quaternary	Change in oligomeric state	Protein–protein interactions Oligomerization	Size-exclusion chromatography Native gel electrophoresis	*	*****	*****

**Table:** 

	Construct design			Expression conditions	
	Removal of degradation-prone and protease sites	Optimized codon usage	Truncations and point mutations	Chaperones and co-expression	Affinity tags for improved solubility
Examples	N-end rule, PEST sequences and specific protease sites	Synthetic gene synthesis offered by many companies	Stabilizing mutations for membrane proteins	Coexpression and fusion vectors with DnaK and GroEL	GST, MBP, Halo tags
Key references	Bachmair *et al.* (1986 ▸), Rogers *et al.* (1986 ▸), Spiegel *et al.* (2015 ▸)	Daniel *et al.* (2015 ▸), Kane (1995 ▸)	Gräslund *et al.* (2008 ▸), Klock *et al.* (2008 ▸)	Kyratsous & Panagiotidis (2012 ▸), Kyratsous *et al.* (2009 ▸)	Walls & Loughran (2011 ▸), Kapust & Waugh (1999 ▸)

**Table:** 

	Expression conditions		Host cells	
	Cofactors and ligands	Low-temperature expression	Codon-optimized cells	Reduced-toxicity and reduced-protease cells
Examples	Metal ions and cofactors essential for folding, also other stabilizing cofactors	Cold-shock, low-temperature inducible promoters such as pCold	*E. coli* Rosetta	*E. coli* BL21 Star and pLysS
Key references	Leibly *et al.* (2012 ▸)	Qing *et al.* (2004 ▸), Vasina & Baneyx (1997 ▸)	Tegel *et al.* (2010 ▸)	Studier (1991 ▸)

**Table:** Many of these techniques, in some way, affect both the *compositional* and *conformational* stability of the protein. Many of these methods have been successfully deployed as so-called ‘salvage’ methods in high-throughput structural genomics consortia.

	Protein crystallizability- and stability-modifying methods				
	Truncations and domain selection	Buffer screening	Ligands and additive screening	Fused affinity tags and crystallization chaperones	Reductive alkylation
*Compositional* stability	Yes	Yes	Yes	Yes	Yes
*Conformational* stability	Yes	Yes	Yes	Yes	Yes
Example	Removal of disordered regions	Stabilize buffers *via* ionic changes	Binding to stabilize protein	Stabilize protein with rigid fusion of tag	Provides entropic benefit upon crystallization
Key references	Yumerefendi *et al.* (2010 ▸), Reich *et al.* (2006 ▸), Klock *et al.* (2008 ▸), Gräslund *et al.* (2008 ▸)	Reinhard *et al.* (2013 ▸)	Chung (2007 ▸), Hassell *et al.* (2007 ▸)	Smyth *et al.* (2003 ▸)	Rice *et al.* (1977 ▸), Tan *et al.* (2014 ▸), Walter *et al.* (2006 ▸)

**Table:** 

	Protein crystallizability- and stability-modifying methods				
	Surface mutagenesis, surface-entropy reduction (SER) and deglycosylation	*In situ* proteolysis	Thermofluor	Deuterium-exchange mass spectrometry (DXMS)	Disulfide engineering
*Compositional* stability	Yes	Yes	No	No	Yes
*Conformational* stability	Yes	Yes	Yes	Yes	Yes
Example	Entropic stabilization of large, flexible, surface-exposed residues	Removal of flexible loops	Analysis of thermal stability (*T*_m_)	Identification of flexible regions	Stabilization of quaternary structure of crystal packing
Key references	Cooper *et al.* (2007 ▸), Goldschmidt *et al.* (2014 ▸)	Dong *et al.* (2007 ▸), Wernimont & Edwards (2009 ▸)	Reinhard *et al.* (2013 ▸), Ristic *et al.* (2015 ▸)	Englander (2006 ▸), Englander & Kallenbach (1983 ▸)	Forse *et al.* (2011 ▸), Quistgaard (2014 ▸)
